# A compendium of Androgen Receptor Variant 7 target genes and their role in Castration Resistant Prostate Cancer

**DOI:** 10.3389/fonc.2023.1129140

**Published:** 2023-03-01

**Authors:** Katie Joanna Miller, Isla Henry, Zoe Maylin, Christopher Smith, Einthavy Arunachalam, Hardev Pandha, Mohammad Asim

**Affiliations:** Department of Clinical & Experimental Medicine, University of Surrey, Guildford, United Kingdom

**Keywords:** AR-V7, prostate, castration, gene, cancer

## Abstract

Persistent androgen receptor (AR) signalling is the main driver of prostate cancer (PCa). Truncated isoforms of the AR called androgen receptor variants (AR-Vs) lacking the ligand binding domain often emerge during treatment resistance against AR pathway inhibitors such as Enzalutamide. This review discusses how AR-Vs drive a more aggressive form of PCa through the regulation of some of their target genes involved in oncogenic pathways, enabling disease progression. There is a pressing need for the development of a new generation of AR inhibitors which can repress the activity of both the full-length AR and AR-Vs, for which the knowledge of differentially expressed target genes will allow evaluation of inhibition efficacy. This review provides a detailed account of the most common variant, AR-V7, the AR-V7 regulated genes which have been experimentally validated, endeavours to understand their relevance in aggressive AR-V driven PCa and discusses the utility of the downstream protein products as potential drug targets for PCa treatment.

## 1 Introduction

### 1.1 AR as the major driver of prostate cancer

**Figure 1:**
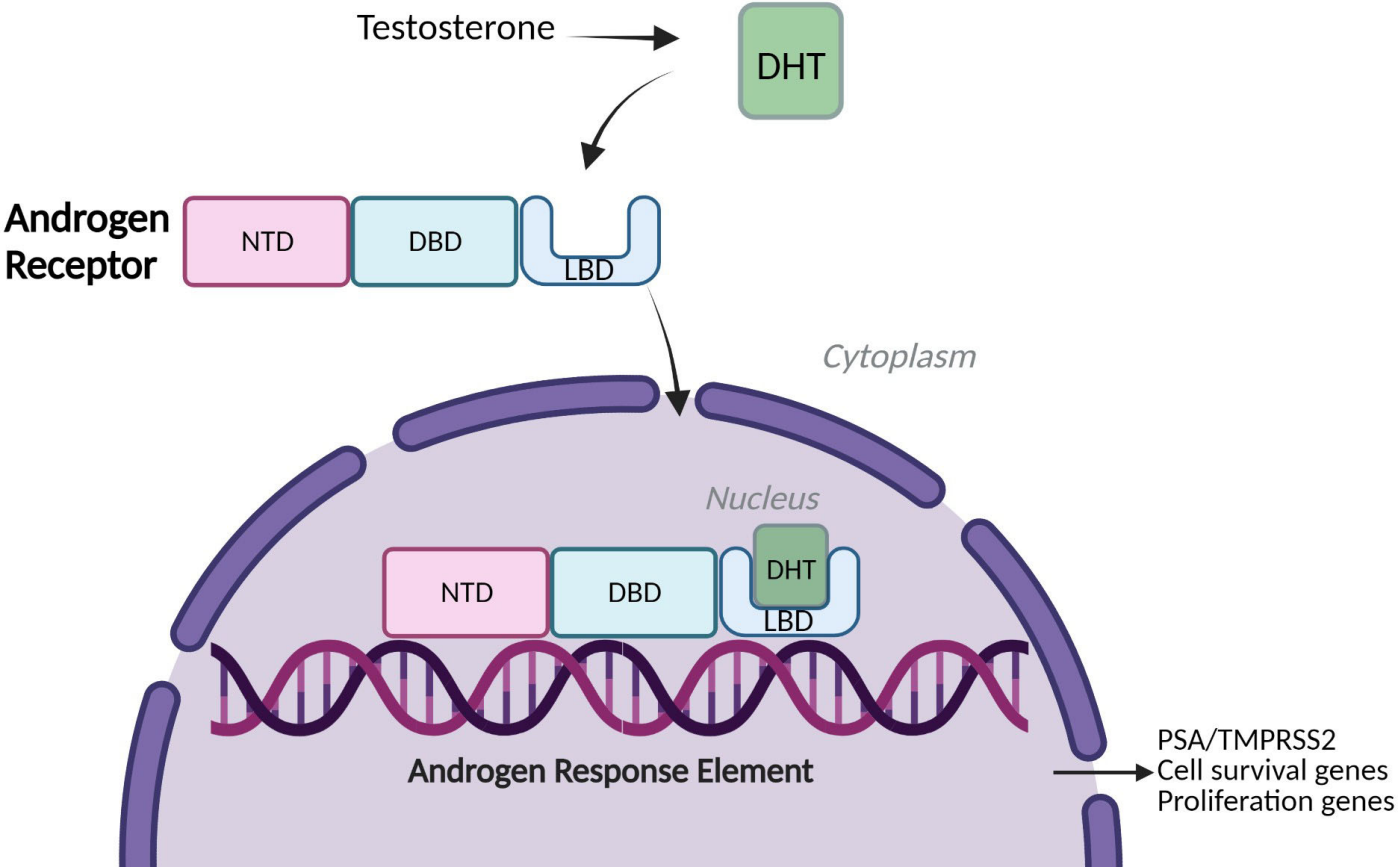
Androgen Receptor signalling. A simplified schematic of Androgen Receptor **(AR)** Signalling: Full-Length Androgen Receptor, a transcription factor binds the testosterone derivative, Dihydrotestosterone (DHT) in the cytoplasm. The AR translocates to the nucleus, binds to Androgen Response Elements (AREs) on DNA and drives expression of cell survival and proliferation genes and target genes such as PSA (KLK3) and TMPRSS2 (2). NTD= amino-terminal domain, DBD= DNA-binding domain, LBD= Ligand-binding domain.

Prostate cancer (PCa) is the second most common cancer in men worldwide, accounting for over 1.4 million new cases every year, leaving one in every six being affected by the disease within their lifetime (1) (2). The prostate is a small walnut-sized gland within the male reproductive system, located underneath the bladder and surrounding the urethra. The growth of prostate epithelial cells is regulated by the actions of androgens which manifest their biological effects *via* the androgen receptor, a member of the nuclear hormone receptor superfamily (3). The AR is a ligand-activated transcription factor (TF) and drives genes associated with proliferation and cell survival along with other target genes such as *KLK3* and *TMPRSS2* (2) (**Figure 1**). Under physiological conditions, AR signalling is responsible for the development and maintenance of the male reproductive system, as well as cell proliferation and apoptosis of prostate epithelial cells (4, 5). However, the uncontrolled overstimulation of AR signalling is considered a major driver of PCa through the regulation of an oncogenic gene signature which supports uncontrolled cell growth. This can further progress to a pre-cancerous condition known as prostate intraepithelial neoplasia (PIN) and subsequent tumour formation (2, 6).

The androgen receptor itself is regulated by a number of transcription factors and related proteins. Transcription factors CREB, Myc, Sp1, Foxo3a, LEF1, NF-ĸB, Twist1, E2F, SREBP-1 have shown to positively regulate AR expression while c-Jun, p53 and Purα demonstrated negative regulation of AR expression. Other transcription cofactors, cytokines, signal transducers and RNA-binding proteins such as β-Catenin, Rb1, Smad2 and 3 and EBP1 also play a role in direct regulation of the AR. Further to this, there are known compounds and agents that alter AR expression by acting on transcription, translation and post-translational activity for example polyphenols acting on hormones and transcription factors (7). It is also well known that the AR and androgens autoregulate AR expression whereby mRNA levels of the AR are unchanged or opposite to AR protein expression levels (8–10).

Given the mitogenic effects of androgens on prostate epithelial cells, the drugs used in the treatment of advanced PCa aim to suppress AR signalling. Androgen deprivation therapy (ADT) either surgically or chemically castrates patients to reduce the levels of free androgens in the blood (2), and thus decrease AR signalling. While ADT is very effective, many patients develop an ADT-resistant, lethal form of the disease known as castration-resistant prostate cancer (CRPC). ADT is often combined with direct AR inhibitors (ARIs) for the treatment of the castration-resistant form of the disease, whereby AR signalling is maintained despite the low levels of androgens. Some ADT drugs such as Abiraterone acetate inhibit adrenal gland enzymes, involved in androgen biosynthesis, and subsequently reduce AR signalling by androgen deficit (2). The majority of AR inhibitors (ARIs), such as Enzalutamide and Bicalutamide, competitively antagonise the binding of androgens to the ligand-binding domain (LBD) of the AR (2, 11). ARIs therefore, reduce the activity of AR signalling often through the inhibition of its nuclear translocation and chromatin binding of the AR leading to the inhibition of AR target gene expression, subsequently causing the suppression of tumour growth (2, 11). ARIs have shown to reduce tumour size and prolong the survival of patients with Castration-resistant Prostate Cancer (CRPC) who had failed chemotherapy (12). Enzalutamide reduces the disease burden as well as prolongs progression-free survival with metastatic hormone-sensitive prostate cancer (mHSPC) (11, 13). However, in metastatic disease, resistance to ARIs usually occurs within 18-36 (14) months due to a transition to CRPC, which has a median survival of 14 months (15). Among others, resistance can occur through several AR centric mechanisms: AR amplification, increased AR promiscuity, increased activation of androgen-independent activation of AR, and AR variants (AR-Vs) (2).

### 1.2 AR Variants

**Figure 2:**
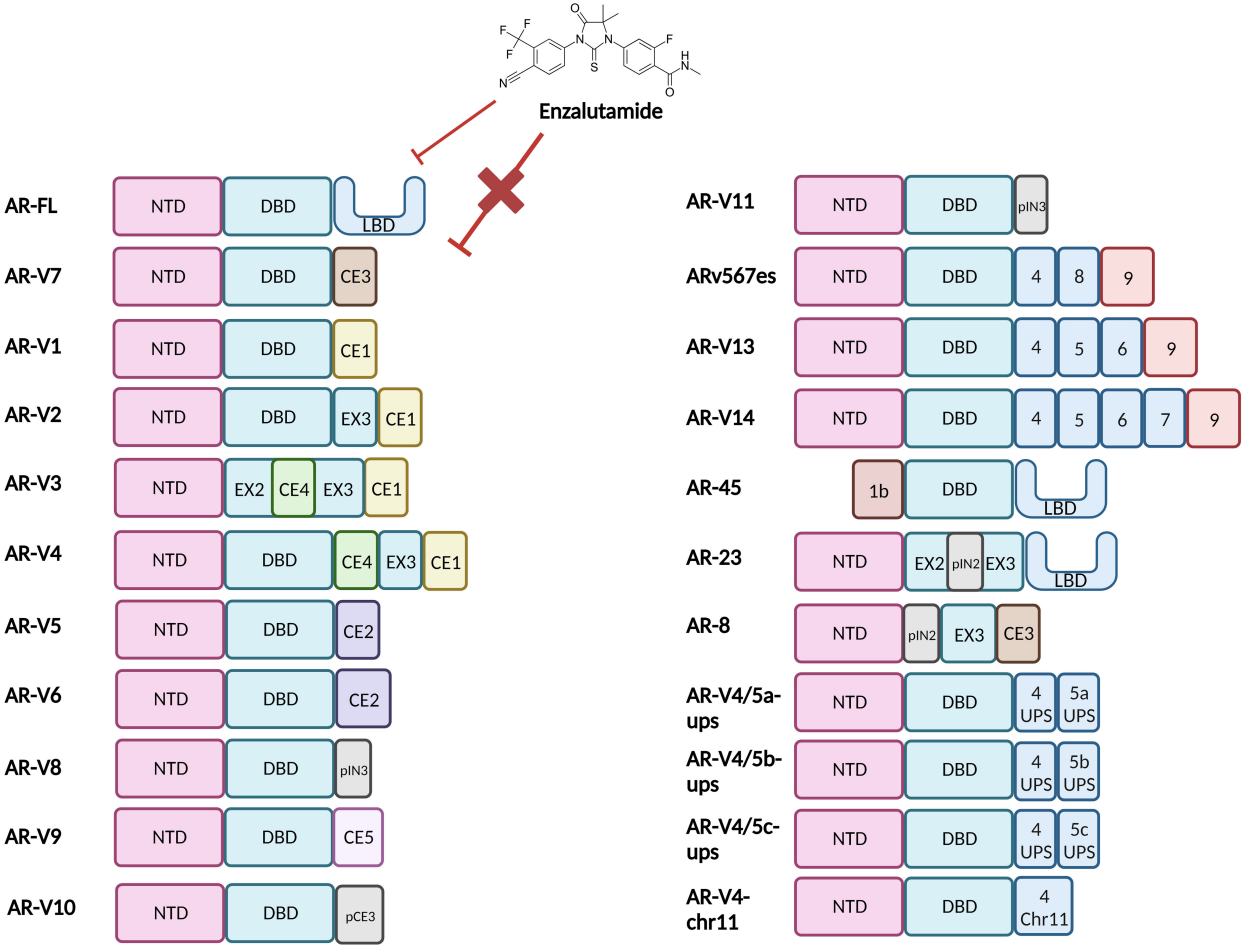
Structural representation of the Androgen Receptor and Androgen Receptor Variants. Full length Androgen Receptor (AR-FL) protein consists of three functional domains: an amino-terminal domain (NTD), DNA-binding domain (DBD) and Ligand-binding domain (LBD). Between the DBD and the LBD is a small hinge region (not shown). The AR gene resides on the X chromosome with 8 exons coding for the protein. Exon 1 codes for the N-terminal domain (NTD), exons 2 & 3 code for the DNA-binding domain (DBD) and exons 4-8 code for the hinge region (HR) and the ligand-binding domain (LBD). The NTD is the primary driver of AR-dependent transcription, the charged DBD allows interaction of AR with the DNA and the hydrophobic LBD facilitates androgen binding. Truncated AR variants (AR-Vs) lack the LBD (e.g. AR-V1 to AR-V14, inclusive of ARv567es) and instead, contain cryptic exons or partial LBD-coding exons or are non-functional and/or carry out alternate functions. In all cases these alterations diminish the ability of the AR to bind to ligands and subsequently be inhibited by LBD-targeting drugs such as Enzalutamide. CE, Cryptic Exon; EX, Exon; IN, Intron; p, Partial.

There are ca. 20 different known AR-Vs which are truncated forms of the full-length AR (AR-FL) (**Figure 2**) (13, 16). Clinically relevant AR-Vs are almost always expressed with AR-FL and may coordinate gene expression *via* heterodimerisation (17, 18). In the context of AR-FL, the LBD represses the AF1 function residing within the NTD when androgens are not bound to the LBD restricting its activation when the androgen trigger is absent (19). However, the lack of a functional LBD results in the NTD of AR-Vs no longer being repressed thus rendering AR-Vs constitutively active. As most clinically employed ARIs target the LBD, AR-Vs represent a treatment escape mechanism and thus their emergence in tumours can predict tumour response to ARI drugs such as Enzalutamide (2). Since AR is known to interact with a wide array of factors that form its transcriptional network, losing the LBD may modulate the AR interactome and could affect its transcriptional output resulting in a change in the AR cistrome, with evidence discussed in this review that there is some differential gene regulation between AR-FL and AR-Vs (20, 21).

The selection pressure of AR-targeting treatments induces genomic rearrangements and/or alternative splicing that form the AR-Vs (22–24). The most researched variant is AR-V7, which is correlated with ARI resistance (25) and reduced survival (26). Sharp and colleagues demonstrated that whilst <1% of primary PCa (hormone naive) had AR-V7, over 75% of ADT-treated tumours show positivity for AR-V7 (25). Similar findings were reported in the SU2C dataset of metastatic PCa (n=125) where 90% tumours showed AR-V7 expression (27). Other variants include AR-V567es, which have a transcriptomic overlap with AR-FL and AR-V7 and may produce an intermediate phenotype whereby target gene regulation and thus aggressiveness of disease is intermediary of AR-FL and AR-V7-driven diseases (28).

There are a few studies that discuss how AR-Vs and specifically AR-V7, are regulated. Hu et al. concluded that AR-V protein expression is negatively regulated by AR-FL signalling (29); Jones et al. determined that Aurora A kinase regulates AR-V7 expression in a feedback loop mechanism (30) while Li et al. showed protein phosphatase-1 and AKT kinase drive AR-V7 expression (31). The alternative splicing that synthesises these AR-Vs thus also contributes to AR-V7 expression where splicing factors have been shown to increase in expression after hormone therapy (2, 32). Interestingly, AR-Vs have been shown to attenuate AR-FL transactivation (33).

### 1.3 AR-V7

AR-V7 is produced in PCa cells as a result of aberrant splicing. It retains exons 1, 2, and 3 which code for the NTD, DBD, and part of the hinge region, as well as the cryptic exon 3 (CE3) that codes for an additional stretch of sixteen further amino acids (**Figure 2**). The splicing of pre-mRNA requires the recruitment of RNA splicing factors to 3’ and 5’ splice sites, which control intron removal and their abrupt functioning can lead to alternative splicing of the mRNA generating AR-Vs (34). Identification of which RNA splicing factors are differentially recruited to the pre-mRNA of AR to induce the formation of different AR-V transcripts may be an avenue for therapeutic targeting outside the realm of posttranslational AR modifications. Liu et al. identified an alteration in splicing factors U2AF65 and ASF/SF2 binding the 3’ site splice site of AR-V7 pre-mRNA under Enzalutamide-induced castrate conditions (23). Miller et al. suggested that SRPK2 may be a key factor in increased availability of ASF/SF2 for this binding, but this requires experimental validation (35).

**Figure 3:**
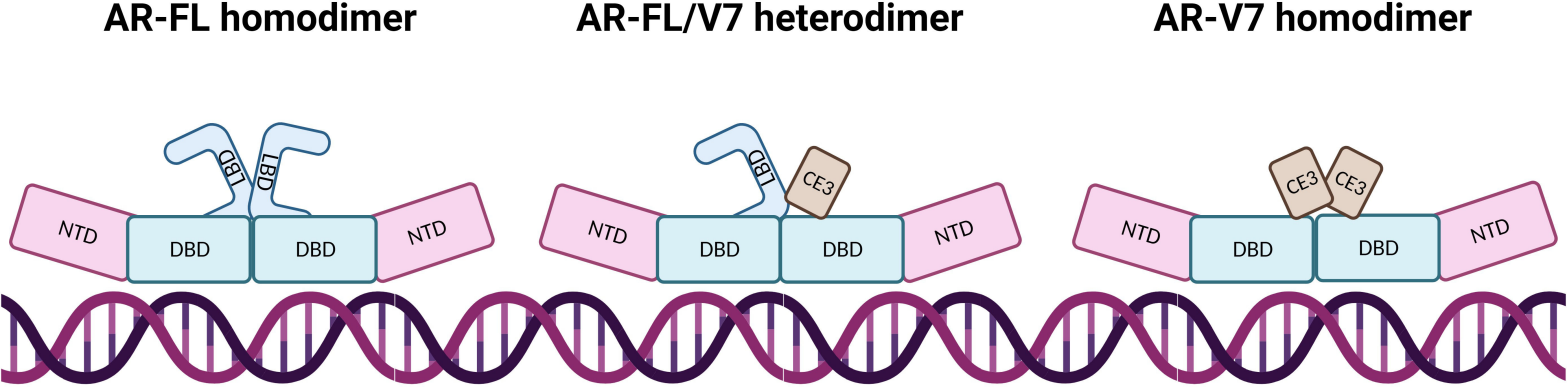
AR dimerisation and interaction with DNA. In AR-FL homodimers, the proteins intermolecularly associate by N/C interaction which is lost after DNA binding, allowing co-activator binding due to NTD and LBD separation. AR-FL/V7 heterodimers interact *via* N/C and D-Box interaction whereby the N/C interaction is also lost upon DNA binding. The AR-FL protein is immobilised on the DNA for a long time whereas AR-V7 protein demonstrates fast DNA-binding dynamics at high-affinity binding sites and dissociates from the dimer shortly after binding. AR-V7 homodimers have a short immobilisation time on the DNA, where dimers have close proximity *via* the DBD.

Constitutive activation of AR-V7 results from the predominant localisation of AR-V7 to the nucleus, and the loss of LBD mediated repression of NTD activation in the absence of hormone binding (25, 36). Other differences between AR-FL and AR-V7 may lie in their interactions with DNA and TFs. In most cases, AR-FL will homodimerise upon ligand activation and bind to DNA. Whilst it is widely agreed that AR-V7 will primarily heterodimerise with AR-FL, the protein can also homodimerise and have more transient DNA interactions (17, 37–39) (**Figure 3**). Özgün et al. discovered that the genomic locations of heterodimers and AR-FL homodimers did not change but their chromatin binding dynamics were independent of each other (17), while AR-V7 homodimer DNA-binding locations have not been fully explored, although it has been determined that AR-Vs bind to constitutively open chromatin sites that are known to overlap AR-FL binding sites (40). Although AR-FL and AR-V7 bind to similar response elements within the DNA, AR-V7 is not simply a constitutively active version of AR but has a unique cistrome, which can be used to experimentally quantify AR-V7 activity. This distinct transcriptomic signature may be explained by different cofactor interactions. AR-V7 exhibits binding to Zinc finger protein x-linked (ZFX) at AR-V7 solo sites (20), increased binding to NCoR corepressors which could alter H3K27ac regulation (37), and a reduced affinity for forkhead box protein A1 (*FOXA1)* (41), which may potentially alter the AR-V interactome.

Since AR-Vs confer resistance to AR inhibition therapies, recent research has been focussing on how to inhibit their activity in CRPC. Targeting of AR-Vs has proven challenging, with new ways of inhibition being required for control in disease progression (2). Novel emerging therapies that target both AR-FL and AR-Vs do not bind and inhibit the AR-LBD. The new treatments belong to four main classes: NTD-targeting, DBD-targeting, AR degraders and AR dimerisation inhibitors. NTD-targeting drugs (e.g. Anitens and Sintokamides) inhibit the AR transcriptional activation while DBD-targeting drugs (e.g. VPC compounds) inhibit AR-DNA interactions. AR degraders (e.g Niclosamide) degrade AR protein by proteasome or non-proteasome-dependent mechanisms to reduce AR protein abundance and AR dimerisation inhibitors (e.g.VPC-17005) impede AR dimerisation and thus interrupts AR interactions with DNA (2).

Successful inhibition of AR-V7 can be experimentally verified by examining the gene expression signature of AR-V7 target genes and therefore, there is a need to identify AR-V7 exclusive target genes. This review will provide a comprehensive account of some of the key AR-V7 target genes and their relevance in ARI-resistant PCa.

## 2 AR-V7 Target genes

**Table 1** summarises AR-V7 target genes and whether they are upregulated or downregulated with AR-V7 expression.

**Table 1:** Summary of AR-V7 target genes defined in the literature.

AR-V7 Target gene		Regulation	References
Abbreviated name	Full name		
EDN2*	Endothelin 2	Upregulated	(21, 28, 41–43)
ETS2*	ETS Proto-Oncogene 2		(37, 41–43)
UBE2C*	Ubiquitin Conjugating Enzyme E2 C		(29, 44–46)
*NPR3*	Natriuretic Peptide Receptor 3		(28, 47)
*SLC3A2*	Solute Carrier Family 3 Member 2		(48)
*NUP210*	Nucleoporin 210		(48)
*BIRC3*	Baculoviral IAP Repeat Containing 3		(21, 42)
*CCNA2*	Cyclin A2		(29, 33, 49, 50)
*AKT1*	AKT serine/threonine kinase 1		(51–54)
*CDK1*	Cyclin dependent kinase 1		(44, 55)
*UGT2B17*	UDP glucuronosyltransferase family 2 member B17		(23, 44, 55)
*CDC20*	Cell division cycle 20		(44, 55–57)
*CDH2*	Cadherin 2		(49, 58)
*SLC30A7*	Solute Carrier Family 30 Member 7	Downregulated	(37)
B4GALT1*	Beta-1,4-Galactosyltransferase 1		(37, 59–61)
*SNX14*	Sorting Nexin 14		(37)
*HIF1α*	Hypoxia Inducible Factor 1 Subunit Alpha		(37)

*Most experimentally promising AR-V7 Target Genes.

### 2.1 Upregulated AR-V7 target genes

#### 2.1.1 EDN2

Endothelin-2 (*EDN2*) was first identified as an AR-V7 target gene in 2014 by Krause et al. The creation of AR-V7 expressing cell lines by transfection with lentivirus containing doxycycline (Dox)-inducible expression of AR-V7 enabled quantification of AR-V7-specific target gene expression by qPCR and therefore identified transcriptional up-regulation of *EDN2* by AR-V7 (41).

*EDN2* encodes the protein endothelin-2 (ET-2) which belongs to a group of secretory vasoconstrictive peptides that are part of the endothelin protein family and is co-regulated by the TF FOXA1 [FOXA1 has been described to co-regulate the expression of many genes with the AR (62)]. The preproprotein (preproendothelin-2) undergoes processing, resulting in a mature form that acts as a ligand for the endothelin receptors. Upon ligand-receptor binding, intracellular signalling cascades leads to vasoconstrictive activity (63). There is a paucity of information in the literature regarding the function of *EDN2* in PCa. However, Grimshaw et al., reported that under hypoxic conditions in breast cancer cell lines, an increase in tumour survival was observed with increases in ET-2 expression levels. The presence of ET-2 has also been shown to increase the migratory and invasive abilities of breast cancer cells (64, 65).

ET-2 receptor antagonists are often used to treat pulmonary arterial hypertension, with approved treatments known as besentan, ambrisentan and macitentan, while other experimental drugs are in the clinical trials (66, 67). The drug tezosentan had been used in clinical trials as an endothelin receptor blocker in the treatment of congestive heart failure. Therefore, there is a potential to repurpose these drugs as a cancer treatment, specifically for AR-V7-expressing CRPC.

*EDN2* expression remains unaltered by synthetic androgen R1881 (41) and is therefore not AR-FL regulated, however there are discrepancies in the literature as to whether *EDN2* is regulated by a wider range of variants than just AR-V7. Several studies have shown that *EDN2* expression can be upregulated by both AR-V7 and AR-V567es (21, 28, 41–43) therefore further investigation is required to identify whether *EDN2* is specific to certain variants or widely regulated by AR-Vs.

#### 2.1.2 ETS2

Alongside *EDN2*, Krause et al. also identified ETS proto-oncogene 2 (*ETS2)* as another AR-V7 target gene (41). ETS2 is a TF that specifically binds the DNA GGAA/T core motif of the Ets-binding site to activate transcription (68). ETS2 regulates genes that are involved in angiogenesis, cell cycle control and cell proliferation, such as plasminogen activator urokinase and c-met (69). Interestingly, ETS2 is known to upregulate the expression of genes involved in prostate carcinogenesis, such as those controlling anchorage-independent growth (68).

There are no known clinically available ETS2 inhibitors, however an experimental molecule has been mentioned in the literature for the treatment of PCa. Carbone et al. designed a triplex-forming oligonucleotide targeting a homopurine:homopyrimidine sequence in the *ETS2* promoter to inhibit transcription (70). This experimental therapeutic holds promise for the treatment of AR-V7-expressing CRPC.

*ETS2* is not an AR-FL target gene as its expression is not induced by R1881 (41), however, whether AR-V7 is not the sole regulator of *ETS2* expression needs further investigation. Studies have demonstrated that upon the induction of AR-V7 expression in cell line models of PCa, *ETS2* expression was upregulated. However, this may not be specific to AR-V7, since when LNCaP were made to express a pan AR-V model, *ETS2* was also upregulated (41–43), indicating ETS2 upregulation may be a general mechanism by AR-Vs.

#### 2.1.3 UBE2C

Ubiquitin-conjugating enzyme 2C (*UBE2C*) was first mentioned as an AR-V gene in metastatic PCa tissue (44) and then later showed a significant correlation between *UBE2C* and AR-V7 expression levels. *In vivo* LNCaP-95CR xenografts treated with Abiraterone showed a significant correlation between *UBE2C* and AR-V7 transcript levels. Immunohistochemical staining also validated this significant correlation (29), indicating UBE2C is a potential AR-V7 target gene.

As a component of the anaphase-promoting complex/cyclosome (APC/C) enzyme, UBE2C is essential for ubiquitination (71, 72). *UBE2C* has been described as a hub gene of PCa i.e., a gene with high connectivity in the genetic interaction network and is involved in many oncogenic pathways involved in PCa progression such as NOTCH signalling and WNT-β-Catenin signalling (71), while also being positively correlated with the progression of other cancers types such as breast and ovarian, whereby its activity promotes a more aggressive tumour phenotype (73, 74). Under normal conditions, the UBE2C protein is necessary for the eradication of mitotic cyclins for the progression through the cell cycle (45, 75). However, in PCa, UBE2C drives cell cycle progression by inactivating the M-phase checkpoint. Alternatively, it can also increase the level of active APC/C (76).

As *UBE2C* is commonly upregulated in multiple cancers, inhibition of this gene has been explored, however inhibitors specifically for this protein are not widely used in the clinic. Bortezomib however is an FDA approved treatment for myeloma and has been shown to also prevent cell cycle progression in Colorectal Carcinoma (77). Therefore, there is potential for this drug to be repurposed as a CRPC treatment. Furthermore, a 2011 study discovered that an mTOR inhibitor, CC1-779 was able to inhibit UBE2C mRNA and protein expression in CRPC cell line models (78), therefore there should be further investigation to fully explore the therapeutic potential of this drug as a treatment for CRPC.

*UBE2C* has often been shown as an AR-V7-regulated target gene (45, 46). Interestingly, studies have shown that in hormone-sensitive PCa (HSPC), there is a significant correlation between the expression of *UBE2C* and the expression of AR-FL. However, as the PCa progresses this correlation shifts to AR-V7 instead of AR-FL, further pointing towards an indispensable role of UBE2C in PCa growth and progression (29, 76). In addition to AR-V7 and AR-FL, AR-V567es can also induce the expression of *UBE2C* and the growth of PCa cells; although this occurs in the presence of mediator complex subunit 1 (MED1) (75, 76).

#### 2.1.4 NPR3

AR-V7 and AR-V567es expressing LNCaP and VCaP cells were generated by Nagandla et al. via lentivirus-mediated transduction. The gene expression mediated by AR-V7 or AR-V567es, relative to variant gene expression in 22Rν1 cells (expressing AR-FL and AR-Vs) was compared to identify differentially expressed genes between the AR-Vs and AR-FL. These data identified Natriuretic Peptide Receptor 3 (*NPR3*) as an AR-V7- upregulated target gene (28).

*NPR3* encodes for natriuretic peptide clearance receptor (NPCR). NPCR represents approximately 94% of natriuretic peptide receptors (NPRs) (79), and removes natriuretic peptides from circulation, thus reducing binding to the other two NPR which have guanylate cyclase activity (80). NPCR has the greatest affinity for the Atrial Natriuretic peptide (ANP) (81), which has been linked to controlling PCa progression (82), therefore an increase in NPCR expression may enhance PCa progression. One of the anticancer effects of long-lasting ANP is the inhibition of phosphorylation and activation of MEK1/2 and ERK1/2 *via* cyclic GMP activation (83, 84), which is required for the activation of AR signalling. This mechanism is also immunomodulatory in the tumour microenvironment of PCa (85). Indeed, the MEK1/2 inhibitor, Trametinib is under Phase II trials for CRPC (NCT02881242) (86). However, as NPCR lacks guanylate cyclase activity, it would competitively inhibit the inhibitory effects of MEK1/2 on ANP thereby reducing the ANP levels in the circulation. However, when ANP binds to NPCR, the oncogenic Vascular Endothelial Growth Factor (VEGF) is inhibited (87). Thus, AR-V7-upregulated NPCR plays an oncogenic role in PCa but can be inhibited by the binding of ANP, which has the potential to be exploited therapeutically to reduce PCa progression.

Inhibition of natriuretic peptide receptors has been investigated in literature, and a few antagonists of the receptors have been identified. Isatin has demonstrated inhibition of atrial natriuretic peptide receptor binding in mammalian cell models (88), while peptides ANP4-23 and AP-811 have shown to be selective atrial NPCR antagonists (89). Along with other experimental molecules, these therapies could have the potential to be used as CRPC therapeutics in a clinical setting.

Further contradiction occurs as to when *NPR3* expression increases in PCa, and if *NPR3* is specifically AR-V7 regulated. AR-V7 regulation of *NPR3* expression, as described by Nagandla et al, would implicate *NPR3* expression is upregulated in CRPC and is corroborated by Terada et al. (47). However, this contrasts with findings of increased NPCR during PIN to PCa transitions (90), and in a CWR22 xenograft model, which is derived from primary PCa and in which AR-V7 expression is rare (91). Furthermore, NPCR was identified in PC-3, a PCa cell line derived from bone metastases (91) which also does not express AR-V7 (36). Therefore, whilst the role of *NPR3* in PCa needs further validation, it is unlikely to be specifically regulated by AR-V7.

#### 2.1.5 SLC3A2

Solute Carrier Family 3 Member 2 (*SLC3A2*) was identified as an AR-V7 target gene by Sugiura et al. following AR-V7-specific ChIP-sequencing investigating LNCaP-95 (PCa cell line expressing both AR-FL and AR-V7) vs LNCaP (PCa cell line expressing AR-FL only), where expression levels were more than two-fold higher in LNCaP-95 (48). Furthermore, the expression of *SLC3A2* was significantly downregulated following AR-V7 knockdown by RNAi assumed to target the AR-V7-specific domain, CE3, indicating it is regulated by CE3-containing AR (AR-V7, AR-V8) (48).

*SLC3A2* encodes for 4F2 heavy chain, aka CD98hc, a subunit of several heterometric amino acid transporters (92, 93). 4F2hc also has a role in glucose availability (94), as well as nucleotide availability to facilitate cell cycle progression (94). The actions on the cell cycle are at least partially due to 4F2hc regulation of other genes including S-Phase Kinase Associated Protein 2 (*SKP2*) (95). Other activities include involvement in integrin signalling (93). Oncogenicity of *SLC3A2* is due to its involvement in amino acid transport adhesion, and proliferation. Indeed, increased *SLC3A2* expression has been observed in several cancers (96–99), including PCa, lung and liver cancers (95). Whilst *SLC3A2* is ubiquitously expressed, its increased expression is linked to poor PCa prognosis as evident from shorter progression-free survival, and association with a higher Gleason score (95, 100).

BCH (2-amino-2-norbornane-carboxylic acid), a non-metabolisable amino acid, has shown to inhibit the disulphide bond-linked heterodimer that consists of CD98hc plus a catalytic light chain (101). This was demonstrated in a panel of breast cancer cell lines, which consequently showed reduced viability and therefore it is conceivable that this inhibition could also be seen in PCa cells and may be used to target AR-V7-expressing CRPC tumours.

Although Sugiura et al. identified the *SLC3A2* gene as specifically regulated by AR-V7 in LNCaP-95, an increase in *SLC3A2* expression by androgen R1881 treatment has been reported which would suggest regulation by AR-FL as well (48, 102). Additionally, *SLC3A2* is also regulated by Activating Transcription Factor 4 (ATF4) (102), another TF for which protein levels are increased upon androgen induction (103). Therefore, *SLC3A2* may be directly regulated by AR-V7, but also indirectly regulated by AR through direct regulation by ATF4. Regulation by multiple TFs indicates the essentiality of SLC3A2 given its vital role in supporting tumour proliferation.

#### 2.1.6 NUP210

Nucleoporin complex 210 (*NUP210*) was also identified as an AR-V7 gene by Sugiura et al., as it was expressed more than 2-fold higher in AR-V7-expressing LNCaP-95 than LNCaP. Specific knockdown of AR-V7 in LNCaP-95 caused a significant decrease in *NUP210* expression. Furthermore, in the TCGA-PRAD dataset, *NUP210* expression positively correlated with AR-V7 expression (48). In line, there was a significant increase in *NUP210* expression in the Grasso dataset (GSE35988) between benign prostate and primary PCa, where there is little AR-V7 expression (48). However, the increase in *NUP210* expression between benign prostate and CRPC was more significant. Whilst AR-FL does directly regulate *NUP210*, AR-V7 appears to have a stronger impact on NUP210 expression.

Functionally, the encoded protein of *NUP210*, also named NUP210, anchors the nuclear pore complex (NPC) - which regulates nucleocytoplasmic transport, although it also has non-structural functions (104, 105). For example NUP210 has been linked to the modulation of gene expression during differentiation (106, 107) and to T cell homeostasis (108). In multiple PCa cell lines, NUP210 has been identified as the regulator of mTOR transport into the nucleus (109), an interactor of AR (110) and plays a role in PI3K-Akt signalling pathways which are often hyperactivated in PCa (111). Nuclear mTOR modulates gene transcription in CRPC to favour proliferation and migration in the absence of AR-FL and is correlated with PCa aggressiveness and disease progression (109).

Direct targeting of NUP210 protein has not been demonstrated, however targeting of the transcriptional and epigenetic regulator, Bromodomian-containing protein 4 (BRD4) with an aminocyclopropenone (ACP) has been explored, ACP-1n has demonstrated inhibition of NUP210 expression in colorectal cancer cells. This is due to NUP210 being a BRD4-driven nuclear complex component (112). Reducing the expression of NUP210 caused a decrease in cancer cell growth and nucleus size, and therefore the application of this inhibitory mechanism in the context of AR-V7-driven CRPC tumours could result in a similar outcome and therefore represent a novel treatment strategy.

#### 2.1.7 BIRC3

Baculoviral IAP Repeat Containing 3 (*BIRC3*), which encodes for Inhibitor of Apoptosis 2 (IAP-2) was first experimentally validated by Basil et al. (21, 42). To identify genes which were differentially bound by AR-FL and AR-V7 on chromatin, Basil et al. utilised LNCaP and VCaP cell lines stably expressing Dox-inducible AR-V7. ChIP-exo was performed using an antibody specific for both AR-FL and AR-V7 isoforms in conditions either recruiting AR-FL and AR-V7 to chromatin or only AR-FL to chromatin. Along with the ChIP-exo data, ChIP-qPCR confirmed that AR-V7 bound with greater affinity to *BIRC3*, and whilst *BIRC3* expression was marginally induced by AR-FL, it was significantly increased with AR-V7 recruiting treatment, indicating that *BIRC3* is positively regulated to a greater extent by AR-V7 than AR-FL (21).

The encoded *BIRC3* protein, IAP-2 has several roles throughout cell death, immunity, inflammation, and proliferation. The role of IAP-2 in evading cell death is not primarily through direct inhibition of caspases (113, 114), but through negative regulation of the necrosome, a protein complex that can carry out both caspase-dependent and independent cell death pathways (113, 115). IAP-2 is associated with the NF-κB pathway, crucial for inflammation and immune responses, in which IAP-2 is required for TNF activation of the NF-κB pathway, but is also a negative regulator of non-canonical NF-κB signalling (114). However, the role of *BIRC3* in cancer is paradoxical, being reported as both a tumour suppressor and pro-oncogenic, even within the same cancer type (116). There is paucity in literature on the roles of *BIRC3* or IAP-2 in PCa, however it has been identified that *BIRC3* is upregulated with a knockdown of Elongation Factor For RNA Polymerase II 2 (ELL2) (117), which in itself is downregulated by AR-FL and AR-V7 (21, 118), and drives proliferation in LNCaP cells (117). Therefore, increased AR signalling reduces ELL2 activity and thus upregulates BIRC3.

The endogenous antagonist of IAPs, second mitochondria-derived activator of caspase (SMAC) has been employed for drug design since the early 2000s and are now in their fourth generation. The fourth-generation optimised non-alanine agonist, ASTX600, showed growth repression in breast cancer mouse models and are now in phase I/II clinical trials (119, 120). Similarly, there at least 8 small molecule IAP antagonists in clinical trials for the treatment of solid cancers including ASTX600, namely: GDC-0152, LCL-161, Debio-1143, Birinapant, GDC-0917 and APG-1387 (119). This pathway has shown it can be inhibited and exploited in terms of cancer treatment, thus giving it great potential to be used to treat AR-V7-expressing CRPC tumours.

Although the data presented by Basil et al. that *BIRC3* is AR-V7 upregulated rather than AR-FL is compelling, *BIRC3* can be regulated by other TFs in PCa. Wang et al. explored IAP-2 protein levels in several PCa cell lines including LNCaP, LNCaP-95 and VCaP (117), but although the latter two contain AR-V7, it was found that LNCaP cells not expressing AR-V7 had the greatest amount of IAP-2. This increase may result from ELL2 downregulation which is commonly observed in PCa whereby ELL2 expression inversely correlates with Gleason scores (121). Furthermore, *BIRC3* has also been identified as a glucocorticoid receptor (GR) regulated gene (122, 123), although this was in the context of bronchial and pulmonary epithelial cells. The GR is also correlated with CRPC and PCa progression (124), whereby androgens can bind the GR to activate proliferation genes due to high homology between the AR and GR (125). Therefore, *BIRC3* is not solely regulated by AR-V7 and crosstalk between the GR pathway should be considered.

#### 2.1.8 CCNA2

Cyclin A2 (*CCNA2*) was experimentally shown to be an AR-V7 target gene in a variety of PCa cell lines including 22Rν1 and C4-2B-ENZ (49, 50). Hu et al. first identified *CCNA2* as AR-V7 regulated by transiently inducing AR-V7 expression in LNCaP cells independent of AR-FL (29). Furthermore, AR-V7 depletion resulted in inhibition of *CCNA2* expression but not when AR-FL was depleted in 22Rv1 cell line (33).

The protein encoded by *CCNA2*, cyclin A2, is a key regulator of both the G1/S and the G2/M checkpoints of the cell cycle through interaction with cyclin-dependent kinase (CDK) 2 and 1 respectively (126), and has further roles in cytokinesis and cell mobility (127). *CCNA2* has been identified as a hub gene for metastasis in PCa (56), and also has significant correlation to reduced overall survival, decreased disease-free survival, and increased biochemical recurrence of prostate cancer (128, 129).

Generally, exclusive cyclin A2 antagonists are much less common, rather inhibitors of its interaction complexes are widely used. Cyclin A2 forms complexes with the cell cycle proteins, cyclin-dependent kinases (CDK1, 2 and 3) and the transcription factor, E2F-1, along with other proteins. Nitrogen-containing bisphosphonates (N-BPs) however, seem to solely target and inhibit the expression of cyclin A2 at the transcriptional and consequently translational level in epithelial cell models (130). RXL peptides have been shown to bind a hydrophobic site on the surface of cyclin A and thus inhibit kinase activity of CDK2, causing cells to undergo apoptosis (131). There are many general cyclin A/CDK inhibitors available commercially such as Indirubin and JNJ-7706621, often targeting the ATP site of CDK (132). Exclusive cyclin A2 or cyclin complex-targeting molecules are important in inhibiting the cell cycle and thus cancer cell proliferation, therefore these inhibitors might be useful for the treatment of AR-V7-expressing CRPC.

It is questionable whether CCNA*2* is solely regulated by AR-V7. Cao et al. demonstrated that *CCNA2* expression did not decrease with AR-FL knockdown or Enzalutamide (33), but an investigational NTD inhibitor EPI-7170 decreased *CCNA2* expression levels. This decrease, however, may not only be due to AR-V7 inhibition but the culmination of multiple NTD-containing AR-Vs in conjunction with AR-V7. In fact, induced expression of AR-V567es in LNCaP also increased *CCNA2* expression (29, 133), although AR-V7 appeared to cause higher *CCNA2* expression (133). Furthermore, *CCNA2* may be regulated by the TF FOXA1 (134), which is also increased in mCRPC (62). Therefore, whilst *CCNA2* expression is not induced by AR-FL, it may be induced by multiple AR-Vs other than AR-V7 as well as FOXA1.

#### 2.1.9 AKT1

The *AKT1* gene encodes a serine-threonine protein kinase and was one of the first AR-V7 genes to be identified. It was the focal AR-V7 upregulated gene in the first paper on AR3 aka AR-V7 by Guo et al. (51). This conclusion was drawn by the culmination of several findings. Firstly, *AKT1* expression decreased following the knockdown of AR-V7 but not following AR-FL knockdown. Furthermore, investigation into the protein levels of the AKT1 kinase showed higher AKT1 protein levels in xenograft tumours of LNCaP transduced with AR-V7 than in the control LNCaP. Finally, ChIP-sequencing for AR-V7 and AR-FL identified that whilst AR-V7 could bind at two sites of the *AKT1* gene, AR-FL did not bind to *AKT1* (51). *AKT1* has since been used as an AR-V7 target gene in multiple cell lines for example, LNCaP treated with Enzalutamide, LNCaP-95, and 22Rν1 to assess AR-V7 expression (52–54).

*AKT1* specifically encodes for AKT1, commonly referred to as protein kinase B, a regulatory kinase implicated in several signalling pathways including PI3K/AKT/mTOR, AR, and MAPK (111). As such, AKT1 is involved in multiple pathways including cell cycle progression and cell survival, and is hyperphosphorylated, which activates AKT1 in over 50% of human cancers (135). Thus, AKT1 inhibition is a treatment option undergoing clinical trials for several cancers, including PCa (111, 136, 137). In PCa, AKT1 is part of several inhibitory feedback loops with the AR (111). AKT1 can also directly regulate AR by phosphorylation of serine 213 in the NTD and serine 791 in the LBD with contrasting outcomes on nuclear translocation and transcriptional activation of AR (138). Despite the generally antagonistic crosstalk between PI3K/AKT pathway, interestingly, AKT1 promotes AR-V7 transcriptional activity (139). Perhaps, therefore, AKT1 can differentially regulate AR-FL and AR-Vs, although this requires experimental validation and is beyond the scope of this review.

AKT inhibitors have been widely discussed in literature as potential anti-cancer therapies, with several clinical trials already taking place for the treatment of mCRPC. Ipatasertib treatment demonstrated a 3-month median survival benefit in a phase II trial and showed consistent results in combination with abiraterone in a phase II trial. Capivasertib and MK2206 are also undergoing clinical trials for PCa (140), while Deguelin, GSK690693, Celebcoxib and Genistein treatment have been explored in pre-clinical studies (141). These AKT inhibitors show encouraging results for the treatment of CRPC and have the potential to become standard therapy in combination with current treatments in the clinic.Whilst *AKT1* is often used experimentally as an AR-V7 target gene, it may also be regulated by other AR-Vs. *AKT1* expression was increased with AR-V expression compared to AR-FL (44), which could be due to multiple AR-V expression. Furthermore, although *AKT1* may not be AR-FL-regulated at the expression level, it is worth considering that the PI3K/AKT pathway can be upregulated by ADT due to inhibitory feedback loops within AR signalling (111), which may indirectly increase *AKT1* expression, independent of AR-V7.

#### 2.1.10 CDK1

Cyclin-Dependent Kinase 1 (*CDK1*) was first shown as an AR-V7 target gene by Jones et al., although had also been previously identified as a hub gene for AR-Vs (30, 44, 55). In 22Rv1 cells grown in ADT conditions, Jones et al. silenced AR-V7 and observed a significant decrease in *CDK1* expression when compared to the control, indicating AR-V7 positively regulates *CDK1* expression.

*CDK1* encoded protein, CDK1 is known as a master regulator of the cell cycle (142–146), alongside having other roles, such as in DNA replication (147), and regulation of several TFs (148–150) including the AR and FOXO1 (151, 152). As a result, increased *CDK1* expression has been linked to the progression of several cancers, including PCa and breast cancer (56, 152–156). One of the mechanisms by which CDK1 promotes PCa is by directly phosphorylating the AR on serine residues 81 and 515, which increases AR transactivation, nuclear localisation, and stability (151, 157–159), thus increased *CDK1* expression will increase oncogenic AR signalling.

As mentioned with inhibition of cyclin A2, CDK inhibitors are widely used to treat cancer. There is however, a well-known inhibitor, RO3306, that is specific to CDK1 and has shown inhibition of the kinase in many cancer models including PCa (160–162). There are no known clinical trials with this drug in mCRPC, but there is scope for this to be used as a prospective treatment for AR-V7-expressing tumours.

Whilst Jones et al. (30) found a significant decrease in *CDK1* expression during AR-V7 knockdown, utilisation of siRNA targeted to AR exon one, which is common to all transcriptionally competent AR isoforms including AR-FL, caused a significantly greater decrease in *CDK1* expression. This indicates that although *CDK1* expression is regulated by AR-V7, it is not specific to AR-V7. Given that Zhang et al. found a significant decrease in *CDK1* expression in cells expressing AR-FL compared to AR-Vs, it appears that AR-Vs are the primary drivers of *CDK1* expression rather than AR-FL (44). Thus, whilst *CDK1* is useful to differentiate AR-FL from AR-Vs, *CDK1* should not be treated as AR-V7 specific.

#### 2.1.11 UGT2B17

UDP Glucuronosyltransferase Family 2 Member B17 (*UGT2B17*) was first described as an AR-V target gene in a study by Liu et al. where metastatic PCa patient samples were collected and split into groups of low and high expression of AR-Vs and then gene profiles of each group were compared (23, 163). This showed that *UGT2B17* was differentially upregulated by AR-Vs (55). Total RNA from human primary and metastatic PCa tissue was quantitatively analysed to find that *UGT2B17* transcript expression was higher in patients with high AR-V expression compared to low expression. Moreover, AR-V-expressing LuCaP 86.2 cells expressed higher *UGT2B17* than LuCaP 35 cells which only expressed AR-FL, thus validating that *UGT2B17* is positively regulated by AR-Vs (44). The correlation between *UGT2B17* and AR-V7 levels was made when VCaP cells were transfected with siRNA targeting AR-V7 and expressed reduced *UGT2B17*, indicating the dependency of *UGT2B17* expression on AR-V7 (23).

*UGT2B17* encodes for an enzyme, UGT2B17, which has an important role in the intermediate step of testosterone metabolism known as glucuronidation. The function of UGT2B17 is to catalyse the transferal of glucuronic acid from UDP-glucuronic acid to steroid hormones, such as testosterone. This catalysation increases testosterone renal excretion, aiding in the maintenance of androgen homeostasis in the prostate (164, 165). In CRPC progression, UGT2B17 may be actively involved in AR signalling *via* increasing c-Src activation, a tyrosine kinase protein normally activated in a ligand-independent manner but also by EGF and cytokine signalling. Therefore, an increase of UGT2B17 protein can increase ligand-independent AR signalling (mainly determined by constitutively active AR-Vs) and androgen insensitivity in castrate conditions by inducing increased testosterone excretion (164, 165) and thus progressing the disease.

Imatinib is known as a selective UGT2B17 inhibitor (166) and has been in phase II clinical trials for hormone refractory PCa in combination with docetaxel (167). There were however issues with toxicity (168) and the trial was terminated. Imatinib has also been purposed as a platelet-derived growth factor receptor inhibitor, however in clinical trials, the treatment proved ineffective for PCa patients (169). Recently however, salicylic acid has been discovered as an uncompetitive inhibitor of UGT2B17 (170). This avenue could be explored for the treatment of AR-V7-expressing CRPC.

Under conditions of androgen deprivation, AR-V7 is necessary for the expression of *UGT2B17* in VCaP PCa cells, but *UGT2B17* is suppressed by DHT-activated AR-FL (23). However, most literature considers UGT2B17 to be a more general AR-V regulated gene. Therefore, further investigation is required to find if this gene is dominantly or solely regulated by AR-V7 relative to other AR-Vs (23, 55).

#### 2.1.12 CDC20

Gu et al. identified that the Cell Division Cycle 20 (*CDC20)* gene was specifically regulated by AR-V7 in CRPC LNCaP-95 cells (56). Following treatment of LBD-targeting Enzalutamide or NTD-targeting EPI-001, *CDC20* expression only decreased with EPI-001 treatment but not with Enzalutamide treatment. The inhibition of AR-FL by Enzalutamide would allow AR-V7 to remain activated in LNCaP-95 cells and drive *CDC20* expression, indicating that persistent expression of *CDC20* following Enzalutamide treatment but not EPI-001 treatment is a result of AR-V7-driven expression of *CDC20* (56). Further studies evaluating the expression level of *CDC20* following AR-V inhibition/knockdown validated *CDC20* to be a target gene of AR-V7 (44, 55, 57).

CDC20 is involved in cell division and associates to the anaphase-promoting complex/cyclosome (APC/C), activating the APC/CDC20 complex (171). The APC/C is an enzyme consisting of multiple subunits and is responsible for mediating the ubiquitin-mediated degradation of securin, the anaphase inhibitor. The onset of anaphase allows the activation of separase, a cysteine protease, which then permits the separation of sister-chromatids during mitosis (172, 173). In CRPC, the abnormal upregulation of *CDC20* leads to the failure of mitotic arrest and progression through the cell cycle and division of cancer cells with aneuploidy (171, 174), thus increasing oncogenic proliferation and driving disease progression.

Inhibition of CDC20 has been demonstrated in multiple cancer cell models including PCa by the compound Apcin (175–177). Mechanistically, it acts as an APC-CDC20 E3 ligase inhibitor, thus interfering with CDC20 binding to its substrates, causing a blockade of mitotic exit and consequently apoptosis. Apcin-based compounds have been developed further to improve antitumour activity, with encouraging results (178). To date, these compounds have not been used in clinical trials, however, they show great potential for the treatment of AR-V7 expressing CRPC tumours.

Whilst some studies have mentioned *CDC20* to be generally upregulated by AR-Vs, none have specified which AR-Vs other than AR-V7 regulate this gene. Thus, further investigation is necessary in order to determine whether *CDC20* is solely AR-V7 regulated (45, 179, 180).

#### 2.1.13 CDH2

Cottard et al. first identified Cadherin-2 *(CDH2)* as an AR-V target gene (58, 181) by overexpressing AR-FL, ARQ640X [an AR-V model (182)] or AR-V7 in LNCaP cells. Cottard et al. discovered that AR-V7 and ARQ640X increased *CDH2* expression while AR-FL induction in these AR mutant models repressed this expression, highlighting differential regulation of AR mutants to AR-FL (58).

The protein product of *CDH2*, N-cadherin is a calcium-dependent cell adhesion protein which allows neighbouring cells to adhere to one another through dimerization of their N-cadherin chains (181). In CRPC, AR-V7 upregulates N-cadherin, however requires DBC1 (deleted in breast cancer 1) as a coactivator to carry out its enhanced transcriptional activities (183). N-cadherin is a mesenchymal cell marker, and often seen increased in expression in many cancers including CRPC during epithelial-mesenchymal transition (EMT) (184). This transition in turn assists with the progression and metastasis of PCa as the cells are able to migrate and invade into other tissues or organs more easily (185, 186).

N-cadherin antagonists have been widely researched over the past three decades and comprise of monoclonal antibody therapy, short synthetic peptides containing the cadherin CAR sequence, tryptophan-containing peptides (12-mers) and synthetic cyclic peptides containing the cadherin CAR sequence HAV (e.g ADH-1) (187, 188). ADH-1 was able to activate apoptosis, inhibit cell migration and demonstrated tumour growth inhibition in pre-clinical animal models and anti-tumour activity in phase I clinical trials for the treatment of solid cancers. Further derivatives of these peptides have been synthesised and tested. ADH-1 has been tested in preclinical AR negative PCa models (189) but further testing in AR-V7-expressing tumours is worth exploring.

*CDH2* is not expressed in HSPC (183) and while Cottard et al. demonstrated that its upregulation is driven by AR-Vs, research has shown and reported *CDH2* to be an AR-V7-specific gene (49, 58, 183).

### 2.2 Downregulated AR-V7 target genes

#### 2.2.1 SLC30A7

Cato et al. identified four AR-V7 repressed genes through several steps (37). First, they used differential expression analysis to identify genes that had increased expression when AR-V7 was knocked down in LNCaP-95. The genes they identified also required an AR-V7 binding site within 50kb of their transcription start site. The expression of these genes was analysed in patient samples and demonstrated significantly different expression levels between CRPC with high AR-V7 expression levels vs low AR-V7 expression levels. These genes were then compared with positively selected genes from a Genome-wide CRISPR knockout screen in LNCaP-95 cells. Four of these genes were found to have a negative effect on CRPC cell proliferation (37). The first of these was Solute Carrier Family 30 Member 7 (*SLC30A7*), which encodes for zinc transporter member 7 (ZNT7) (37).

ZNT7 transports zinc from the cytoplasm into the Golgi apparatus for storage and incorporation as a cofactor into alkaline phosphatases (190, 191), and was found *in vivo* to be important for dietary zinc absorption and body fat accumulation (192). Whilst a null-mutation of *SLC30A7* in the Transgenic Adenocarcinoma of Mouse Prostate (TRAMP) mouse model of PCa accelerated prostate tumour formation (193), the role of *SLC30A7* in CRPC has not been extensively studied. Zinc deficiency however, has shown to induce DNA damage in normal human prostate epithelial cells (194), and thus downregulation of ZNT7 by AR-V7 could indicate an altered DNA damage response facilitating increased genomic instability.

ZNT7 agonists or stimulators have not been developed and it is unlikely that upregulating this protein is possible for the treatment of AR-V7 expressing CRPC tumours. Current literature does however show that hyperglycaemic states can increase ZNT7 protein expression (195). The mechanism behind this could be explored for potential drug treatments, however caution should be given as hyperglycaemia can have many other health implications for patients and this mechanism therefore may not be clinically relevant.

There are conflicting findings in the literature around *SLC30A7* activity and expression in PCa. Firstly, Cato et al. found *SLC30A7* expression was lower in PCa patients that developed metastasis vs patients that did not develop metastasis (37). This was supported by Tepaamorndech et al. who showed a null-mutation of *SLC30A7* caused acceleration of prostate tumour formation (193). However, a reduction in *SLC30A7* expression would imply that alkaline phosphatases would decrease in activity, however the enzyme alkaline phosphatase is a serum-biomarker of bone metastasis in PCa (196), an event in which AR-V7 is highly expressed (55). Moreover, alkaline phosphatase activity was found to be increased in the metastatic epithelial ARCaPM PCa cell line in comparison to the paired and non-metastatic ARCaPE mesenchymal PCa cell line (197). However, alkaline phosphatase levels remain high in many cancers and the high levels in serum may be produced elsewhere, while the metastatic mesenchymal PCa cell line is not fully representative of the majority of mCRPC which are derived from epithelial cells. More experimental validation is required to identify the role of *SLC30A7* in PCa, and to confirm *SLC30A7* as an AR-V7 regulated gene.

#### 2.2.2 B4GALT1

The second of the AR-V7 target genes Cato et al. identified was β-1,4-galactosyltransferase 1 (*B4GALT1*) (37). B4GALT1 is an important enzyme in the post-translational modification of proteins such as PD-L1 through the formation of complex glycans (37). Indeed, galactose is involved in the synthesis of the core O-linked glycans and in some N-linked glycans, and dysregulation of glycosylation occurs in many cancers including prostate cancer (198, 199). *B4GALT1* has increased expression in several cancers including glioblastoma, breast cancer, and leukaemia (200–203), but shows decreased expression in colorectal, endometrial, and metastatic prostate cancers (59, 60, 204). A conflicting report by Radhakrishnan however, states upregulation of B4GALT1 can be seen in PCa by TNFα stimulation to increase motility and invasiveness of the disease (205) and multiple reports show upregulation of B4GALT1 increases drug resistance in cancers (206–209).

There are no known B4GALT1 agonists, however one study found upregulation of B4GALT1 at the mRNA level by lectin stimulation (specifically Concanavalin A) (210). This was however, demonstrated in immune cells and therefore investigation into whether this would translate in PCa cells should be explored. There is potential, however if this mechanism can be proved in PCa, for Concanavalin A to upregulate B4GALT1 and be used as therapy for AR-V7-expressing CRPC.

Wang et al. found that *B4GALT1* was downregulated by both Aldo-keto Reductase Family 1 member C3 (AKR1C3, an androgenic enzyme) and AR-V7, indicating a complex formed by these two proteins, transcriptionally repress *B4GALT1* (59–61) and allows tumour progression. This complex may explain why Al-Obaide et al. (207) did not identify any AR binding sites in the *B4GALT1* promoter. AKR1C3 can also bind and colocalise with AR-FL, however this phenomenon is often seen with ADT when AKR1C3 expression is higher to promote CRPC development (211). This downregulation of *B4GALT1* could reduce NOTCH signalling due to the reduced galactose activity (212) and thus inhibit the tumour suppressor activity of NOTCH signalling such as reducing the innate and adaptive immune response or reducing the p53 checkpoint protein (213). Although *B4GALT1* is an AR-V7 repressed gene, the extent to which it may be regulated by AR-FL while lesser, is not fully understood. Furthermore, the repressive effect of other AR-Vs on *B4GALT1* has not yet been investigated. Despite this, *B4GALT1* is the most promising AR-V7-repressed target gene.

#### 2.2.3 SNX14

Sorting Nexin 14 (*SNX14*) is the third gene that was found downregulated by AR-V7 by Cato et al. (37) and encodes the protein, SNX14, which is primarily involved in intracellular trafficking. SNX14 binds to phosphoinositides on endosomal membranes for trafficking to the endoplasmic reticulum, and also plays a role in lipid metabolism in the endoplasmic reticulum (214, 215), in the nervous system and in bone metabolism (216, 217). SNX14 deficiency has been linked with an upregulation of proinflammatory cytokines such as TNF and IL-1β (218). TNFα as previously stated induces PCa migration to induce metastasis while IL-1 has also been positively correlated with bone metastasis, and thus this is a possible mechanism by which SNX14 downregulation drives the progression of PCa (219, 220).

SNX14 agonists or upregulators have not been widely explored. However, one study showed that protein kinase A-mediated phosphorylation of SNX14 inhibited its activity (221) and therefore PKA inhibitors could increase SNX14 in cells. Furthermore, another study demonstrated that the release of miR1324 inhibited expression of SNX14, therefore a degrader of miR1324 could upregulate SNX14 expression. However, considering the role that SNX14 plays biologically, manipulation of these processes may be a risk to patient health.

In PCa, the genomic region chromosome 6q14-q22, which contains *SNX14*, is commonly deleted (222), and therefore suggested by Dong et al. to contain at least one tumour suppressor gene. The genes within this region were identified *via* deletion mapping and did include *SNX14*. *SNX14* was removed from the analysis as Dong et al. stated that *SNX14* expression was not detected in normal prostate and concluded that *SNX14* did not have a role in the normal function or the structure of the prostate (222). However, Cato et al. and the NCBI gene database do show that there is expression of *SNX14* within normal prostate (37, 223), thus investigations into SNX14 and its role in PCa and AR-V7 driven disease could be reopened for insight into SNX14 as a target gene.

#### 2.2.4 HIF1A

Hypoxia Inducible Factor 1 Subunit Alpha (*HIF1α*) is the last of the four AR-V7 downregulated genes identified by Cato et al. HIF1α is the alpha subunit of the HIF-1 TF and plays a large role in regulating the cellular homeostatic response in the event of hypoxia, through the transcriptional initiation of genes involved in angiogenesis, apoptosis, and increasing oxygen transportation (37). Given the role of *HIF1α*, it is generally considered an oncogene and has indeed been identified with promote cancer progression in several cancers such as pancreatic cancer, colon cancer and renal cell carcinoma (224–226). In PCa, the overall literature also points to HIF1α’s role as an oncogene, and is seen overexpressed in CRPC (37, 227–230). Furthermore, it is known that AR-FL and AR-V7 can heterodimerise with HIF1α to drive transcription (231) and therefore it is expected that AR and HIF1α transcript levels would correlate, which is in contrast to the findings of Cato et al.

Hypoxia-mimetic agents are well known HIF1α activators used to treat ischemic stroke (232). Iron chelators such as Deferoxamine mesylate (DFO), HIF-prolyl hydroxylases (PHD) inhibitors such as FG-4497 and the antioxidant, N-acetylcysteine treatment have all shown to increase HIF1α expression *via* various mechanisms. There is potential in using these agents to treat AR-V7 expressing CRPC, however due to the conflicting reports in literature often stating HIF1α upregulation in many cancers, this may not be the ideal strategy for treatment.

Tran et al. performed immunohistochemical staining of HIF1α on CRPC tumour biopsies, revealing that there were high levels of HIF1α expression present in CRPC (229). Zhong et al. confirmed an overexpression of HIF1α in prostate adenocarcinomas compared to normal prostate tissues (230). The overexpression of HIF1α under normoxic conditions has been shown to promote the progression of CRPC (233), and Carnell et al. have suggested that a hypoxic microenvironment within the prostate is a primary contributor to secondary genetic alterations, which result in enhanced malignancy and a more aggressive PCa phenotype (234, 235). Therefore, due to the collection of literature contrary to the downregulation of HIF1α in PCa metastasis and its potential role as a tumour suppressor, we do not consider HIF1α a promising AR-V7 target gene. HIF1α is often seen activated in larger hypoxic tumours, and thus we hypothesise that in tumours with decreased tumour burden often seen immediately after Enzalutamide treatment, there may not be an upregulation of HIF1α due to the absence of hypoxic environments and thus why we see HIF1α downregulation as reported by Cato. Furthermore, it was discovered recently that FOXA1 can bind and repress HIF1α, where this complex regulates macrophage invasion and PCa cell invasion and thus driving disease progression. Therefore, increased FOXA1 co-expression with the AR may contribute to this HIF1α repression (236).

## 3 Discussion

Based on the available research, it appears that the majority of the AR-FL regulated genes are also co-regulated by AR-Vs, presumably due to: the co-existence of both forms in a majority of CRPC tumours; their similar structure; and the fact that they can heterodimerise to drive transcription (21, 28, 37, 237). Thus, defining sole AR-V7 targets to study its transcriptional activity is challenging. Additionally, other TFs such as the glucocorticoid receptor (28) or other AR interactors may also alter AR signalling and gene expression (35, 62, 238, 239). Furthermore, research discussed in this review used AR-V models experimentally before validating AR-V7-specific regulation. The truncated AR models used lack the LBD, but do not contain the cryptic exons that most variants additionally contain, therefore these models could be broader models encompassing most of the AR-Vs, and therefore it is possible that genes validated as AR-V7-regulated may also be regulated by other AR-Vs since there is not yet the evidence to prove otherwise.

To verify these genes as AR-V7 target genes, analysis can be carried out using publicly available datasets, however this is beyond the scope of this review, although this review does use all available literature that discusses AR-V7 target genes in the studies carried out. The ideal dataset would be a large RNA-seq study with patient samples, some of which have CRPC and available AR-V7 expression data. However, there is a deficit of such datasets; on cBioPortal there are 8 datasets for “prostate cancer” that have RNA-seq data, and the only one with CRPC samples is the SU2C dataset (27, 240). Although AR-V7 data is also available in the TCGA Cell 2015 dataset, only benign prostate and primary PCa tissue are represented, with AR-V7 only present at very low levels in 42 of the 333 samples (241). On the GEO, there are 29 returns for “prostate cancer” with “AR-V7” and “Expression profiling with RNA-seq”, and whilst the majority of these assess novel inhibitors, 2 contain a small number CRPC patient-derived samples and 10 directly assess the impact of AR-V7 inhibition in PCa cell lines with siRNA, antibody, or shRNA (**Table 2**). However, the small number of clinical samples and the cell lines will not be as representative of most patients as the large clinical SU2C dataset.

**Table 2:** Datasets that can be utilised to determine AR-V7 target genes.

Dataset Name	Sample Type	Direct inhibition of AR-FL or AR-V7
SU2C/PCF Dream Team, Cell 2015 (27, 240)	Patient samples	None
GSE149433 (242)	Patient derived xenograph models, LNCaP-95	None
GSE56701 (26)	Patient metastatic tumour	None
GSE184676 (21)	LNCaP-95	siRNA for AR-FL or AR-V7
GSE158556 (243)	LNCaP-C42B-MDVR	Three antibodies which inhibit AR-FL, AR-V7, or both
GSE171045 (244)	LNCaP-95	AR-FL depletion
GSE143905 (21)	LNCaP, VCaP	Cell lines lentiviral-induced AR-V7 expression with Doxycycline treatment.
GSE122923 (48)	LNCaP, LNCaP95	shRNA for AR-V7
GSE158557 (245)	LNCaP	Cell line lentiviral-induced AR-FL or AR-V7 expression with Doxycycline treatment.
GSE137833 (246)	VCaP, LNCaP, LNCaP-Enzalutamide-Resistant, CRISPR-knock-in AR-V7 LNCaP	AR-FL depletion
GSE94013 (20, 247)	22Rν1	shRNA for AR-FL or AR-V7
GSE99378 (248)	LNCaP-95, 22Rν1	siRNA for AR-V7
GSE71334 (249, 250)	LNCaP	Cell line lentiviral-induced AR-FL or AR-V7 expression

Practically, target gene expression is analysed to determine whether drugs have inhibitory activity, and in this case to determine whether drugs can inhibit AR-Vs lacking a complete LBD. Therefore, it is recommended that target genes that are used experimentally should have a distinction between regulation by AR-FL and AR-Vs and as little regulation by other TFs. From our literature search, we deduce the most promising AR-V7 target genes for experimental use are *UBE2C*, *EDN2*, *ETS2* and *B4GALT1*, as these have been thoroughly validated from multiple sources and show the least regulation from other TFs.

A few other genes such as *OLAH*, *SDC1*, *SRD5A1*, *ORM1*, *HSP27, C-MYC* and *HES1* (21, 29, 42) have been discussed in literature as also being AR-V7 target genes, however there is not yet enough evidence to be certain that these are true AR-V7 target genes. Furthermore, Lu et al. reviewed several studies (251) that discuss AR-V-regulated genes, including research from Guo et al. and Li et al. Guo listed genes “preferentially regulated” by AR3 (AR-V7) after data was collected during overexpression of AR-V7 in LNCaP cells such as *WNK1* and *ADCY6* (51) while Li compiled a list of AR-V upregulated genes after siRNA targeting of exon 1 and 7 of the AR including genes such as *NPTX* and *APOD* (133). It is questionable however, whether these are also AR-FL or general AR-V regulated and therefore further validation is needed to prove whether these are AR-V7 specific.

Moreover, there are a few other interactors of AR-V7 that should be investigated further. *FOXA1* as previously mentioned, is a transcription factor that is positively correlated with CRPC, and is found to co-regulate genes with the AR (62). Krause et al. discovered that there was differential co-regulation of genes with *FOXA1* when AR-FL was present vs when AR-V7 was present. They showed that previously mentioned genes *EDN2* and *ETS2* that are initially negatively regulated by AR-FL are then positively regulated by AR-V7 (41). Therefore, there is scope to further explore additional genes that follow this pattern. Moreover, the *HOXB13* gene encodes a homeodomain-containing TF that is important for normal prostate development and function, and it is primarily expressed in the nuclei of luminal epithelial cells within the prostatic epithelium (252, 253). Elevated expression levels of *HOXB13* have been shown in CRPC tumours when compared to hormone-sensitive tumours (253), and it has been demonstrated that HOXB13 interacts with the DBD of the AR, regulating the transcriptional activity of genes containing AREs (254). Furthermore, recent evidence has emerged demonstrating that HOXB13 mediates AR-V7 genomic-binding activity, and that silencing HOXB13 decreased CRPC growth both *in-vitro* and *in-vivo* (248, 255). These results highlight an interesting avenue to explore in terms of the discovery of new AR-V7 target genes that are coregulated by *HOXB13*.

**Figure 4:**
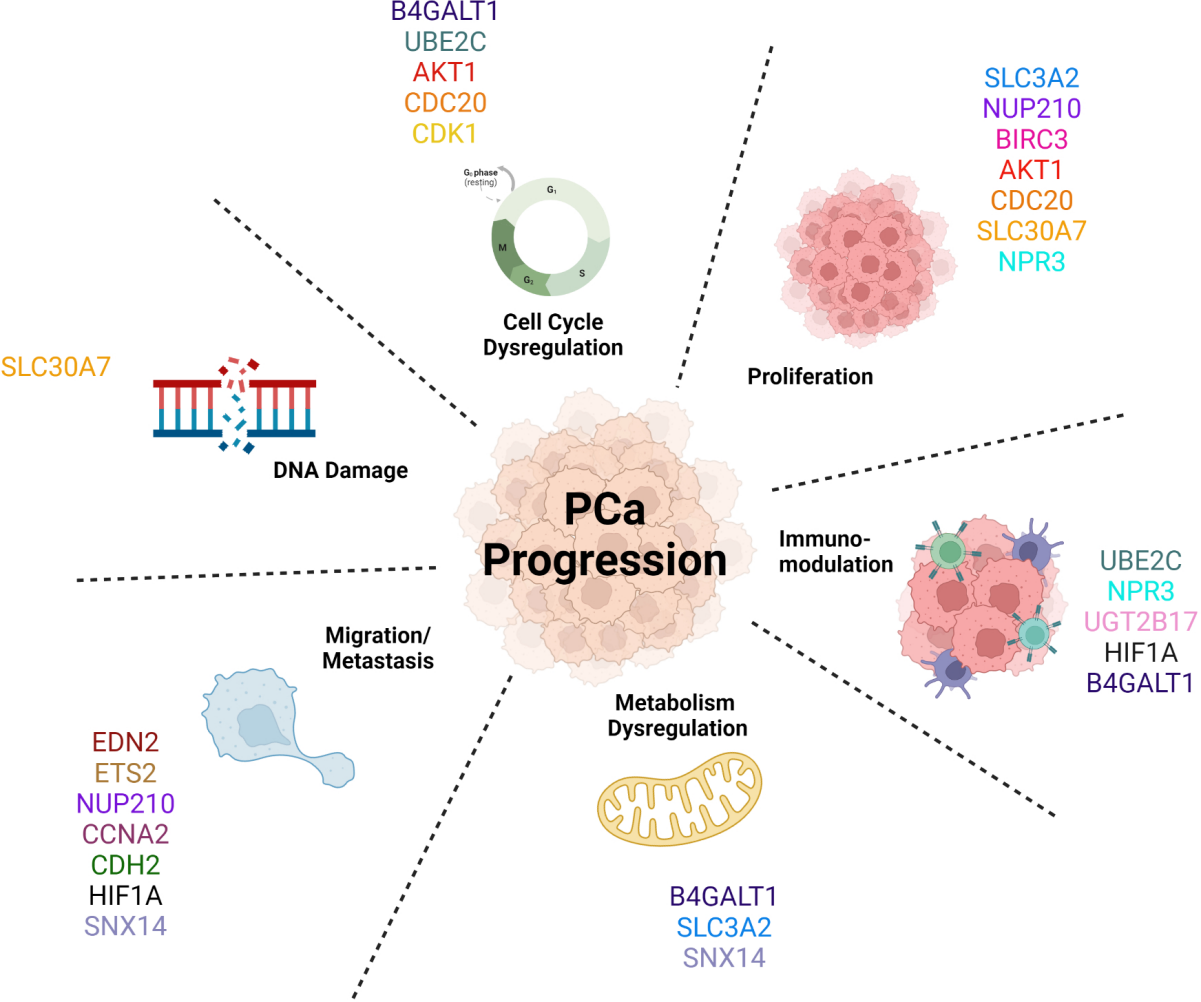
AR-V7 target genes promote Prostate Cancer progression by driving a more aggressive phenotype by activating multiple biological pathways.

The AR-V7 target genes mentioned in this review are linked with a more aggressive phenotype of PCa (**Figure 4**), aligning with clinical evidence concerning prognosis and diagnosis; whereby if a patient’s cancer expresses these truncated AR-Vs, the patient is more likely to have a more aggressive form of PCa, compared to patients with tumours only expressing AR-FL (26, 256). These target genes are involved in and drive many biological pathways that determine this more aggressive phenotype, for example the overexpression of *AKT1* drives cell cycle progression and cell survival of these cancer cells thus increasing cell proliferation (135). Moreover, *CCNA2’s* role in metastasis means that its upregulation will increase cell migration of tumour cells and thus increase the likelihood of secondary tumour formation (127). As AR-V7 decreases the activity of the protein encoded by *SLC30A7* that transports zinc (190), this could cause more DNA damage and impair DNA repair and thus allows new mutations in cancer, causing disease progression (194) (257),. To summarise, AR-V7 is responsible for the significant upregulation of several cancer hallmark pathways. Sugiura et al. claimed the epithelial mesenchymal transition (EMT) pathway, which plays a role in migration and metastasis, to have been the most significantly upregulated by AR-V7 in CRPC (48, 258). However, there is a lack of information in the literature to make claims about the most prominent pathway that is regulated by AR-V7.

The fact that AR-Vs promote a more aggressive phenotype highlights the need for new therapeutics to inhibit them since the antiandrogen drugs currently in the clinic only target AR-FL. Many experimental drugs are currently being tested to determine their efficacy, such as those named in the review by Maylin et al. (2) and thus exploring the expression of the mentioned target genes in PCa models under these novel drug treatments can be a helpful experimental tool. Furthermore, there is also scope for targeting the protein products of the AR-V7 target genes, with a plethora of options available, summarised in **Table 3**, which have the potential to inhibit growth of AR-V7-expressing CRPC cells.

**Table 3:** Druggable strategies targeting protein products of AR-V7 target genes.

Gene	Protein product	Potential therapeutics	References
*EDN2*	ET-2	Besentan, ambrisentan, macitentan	(66, 67)
*ETS2*	ETS2	Triplex forming-oligonucleotide	(70)
*UBE2C*	UBE2C	Bortezomib, CC1-779	(77, 78)
*NPR3*	NPCR	Isatin, ANP4-23, AP-811	(88, 89)
*SLC3A2*	CD98hc	BCH	(101)
*NUP210*	NUP210	ACP-1n	(112)
*BIRC3*	IAP-2	ASTX600, GDC-0152, LCL-161, Debio-1143, Birinapant, GDC-0917, APG-1387	(119, 120)
*CCNA2*	Cyclin A2	N-BPs, Indirubin, JNJ-7706621, RXL peptides	(130) (131, 132)
*AKT1*	AKT1	Ipatasertib, Capivasertib, MK2206, Deguelin, GSK690693, Celebcoxib, Genistein	(140, 141)
*CDK1*	CDK1	Indirubin, JNJ-7706621, RO3306	(132, 160–162)
*UGT2B17*	UGTB17	Imatinib, Salicylic Acid	(166–168, 170)
*CDC20*	CDC20	Apcin	(175–178)
*CDH2*	N-cadherin	Monoclonal antibodies, short synthetic peptides, tryptophan-containing peptides, synthetic cyclic peptides	(187, 188)
*SLC30A7*	ZNT7	n/a	
*B4GALT1*	B4GALT1	Concanavalin A	(210)
*SNX14*	SNX14	n/a	
*HIF1*	HIF1α	Iron chelators, PHD inhibitors, N-acetylcysteine	(232)

## Author contributions

KM and IH wrote the majority of the manuscript with planning, direction and editing from ZM. ZM and CS wrote the discussion. ZM and IH prepared the figures. EA, CS, HP and MA further edited the manuscript and provided guidance throughout. All authors contributed to the article and approved the submitted version.
